# The Institutional Library

**Published:** 1918-06-01

**Authors:** 


### THE INSTITUTIONAL LIBRARY.

**A Manual of Physics for Medical Students.** Second Edition. By HUGH C. H. CANDY, B.A., B.Sc. (London: Cassell and Co., Ltd. 1918. Pp. 451+viii. Price 7s. 6d. net.)

MR. CANDY'S manual has come through the test of time with a considerable measure of success, and it now appears in an enlarged and second edition. It cultivates a limited objective—namely, the instruction of medical students in physics to such a level that they may be fitted for the test of the Preliminary Scientific Examination. One of the great merits of the book is that it keeps this limited purpose consistently in view. So many writers of text-books for students appear to forget the difference between an elementary hand-book and an exhaustive treatise that Mr. Candy's steady resolution merits recognition. It is the custom of some to scoff at books of this order, but for our part we believe that amongst the most useful contributors to the spread of knowledge are the writers of small, sound text-books in which the foundations of scientific subjects are clearly defined and explained. Such a method conduces to accurate mental perspective and secures the ground plan for all without limiting the activities of the student who has time and inclination for cultivating a more detailed knowledge. To wise judgment in the selection of his material Mr. Candy adds the gift of clear exposition and a firm belief in the value of illustrations. Thus his text-book is admirably adapted to the constituency it is meant to serve, and we are glad to give it a word of hearty good-will.

**The Fitting Out and Administration of a Naval Hospital Ship.** By EDWARD SUTTON, Fleet Surgeon, R.N. (London: J. Wright and Sons. 8s. net.)

FLEET-SURGEON SUTTON is well qualified to write with authority on hospital ships. He served as surgeon in the R.N. hospital ship *Malacca*, and has been S.M.O. in the hospital ships *Drina* and *Plassy*. As his service in the two latter ships has been almost for the entire duration of the present war he has had unusual opportunity for studying the subject under war conditions. The book is divided into four sections. Section I. deals with the evolution of the hospital ship from the year 1690 onwards, and also explains the Geneva Convention as applied to hospital ships. Section II. describes the ss. *Drina* and her conversion into a Royal Naval hospital ship. Section III. deals with the ordinary organisation of a hospital ship, and Section IV. with emergency organisation (the admission of more than the normal complement). There are also appendices giving the various forms and vouchers in use for carrying out routine duties. The most valuable part of the book for present use and future guidance is naturally found in the last three sections. The author speaks with evident approval of the specifications provided by the Medical Department of the Royal Navy for the accommodation required and also for the various accessories. As evidence of the completeness of these specifications it is interesting to note that the ss. *Drina*, which arrived at Liverpool on August 4, 1914, was converted into a fully-equipped hospital ship in ten days. While, however, giving general approval to the arrangements made by the Admiralty, the author gives valuable hints derived from practical experience. The description of the accommodation and fittings of R.N.H.S. *Drina* is very minute. The dimensions are given not only of the wards, but even of cupboards, pantries, tables, ice-chests, etc., and details are given of the numbers of towel-hooks, looking-glasses, and various other fittings, besides minute particulars of ventilation and lighting. Some of these details may seem superfluous to the landsman, but are important to the sailor who has learnt to make use of every inch of space which the limited accommodation on board ship provides.

Section III. deals with the ordinary organisation as arranged by the S.M.O. himself. This appears to have been carried out very much on the lines of a naval hospital ashore, the medical officers all meeting in the duty cabin at 9 A.M. and being told off for their duties for the day. An ingenious system is described for registering the exact distribution of the sick-berth staff at any particular moment. Interesting details follow of embarkation and disembarkation routine, and there are excellent descriptions and illustrations of various forms of stretcher and cot.

The book will be a valuable record in future of how a hospital ship has been run under war conditions and is of present value to all concerned in the designing and administration of these ships. It would have been more complete could details have been given of actual experiences and the difficulties and emergencies which have arisen on active service. We should have liked to know how hospital ships have fared during and after naval actions, the number and nature of the cases admitted on these occasions, and the position of the hospital ship in relation to a fleet in action. Probably these details are not allowed to be published at present, but they would make a very interesting supplement "after the war."

**Surgical Applied Anatomy.** By SIR FREDERICK TREVES, Bart., G.C.V.O. Seventh Edition. Revised by ARTHUR KEITH, M.D., LL.D., and W. COLIN MACKENZIE, M.D., F.R.C.S. (London: Cassell and Co., Ltd. Pp. 702 x x. Price 10s. 6d. net.)

SINCE its first appearance in 1884 this book has been so widely known among medical students that the great majority of present-day practitioners must, we imagine, have it on their shelves. Its original plan and execution received prompt recognition as admirably adapted to the purpose it was intended to serve, and they remain to-day substantially unchanged. Revision in details has been practised in successive editions as these have made their appearance, but the lines of the original enterprise have needed but little modification. Altogether it is an admirable piece of work, and well adapted to teach the student the application of anatomical knowledge to the purposes of medical craftsmanship. In addition to a critical survey of the whole book the revisers of the present issue have very appropriately enlarged the chapters which relate to the treatment of stiffened joints and disabled limbs, for the war has multiplied opportunities, or rather necessities, in these directions. What has happened in the past will doubtless be repeated in the future; that is, the book will go from success to success and will continue to be recognised as an essential volume in every medical student's library.

**Married Love: A New Contribution to the Solution of Sex Difficulties.** By MARIE CARMICHAEL STOPES, D.Sc. (London: A. C. Fifield, 13 Clifford's Inn, E.C. 4. Price 5s.)

To say that a book ostensibly upon this subject is not a fraud upon its purchasers is to give it high praise. For

**THE INSTITUTIONAL LIBRARY**—(*continued*).

it is hardly too much to say that the full significance of marriage has been lost, and that its natural laws and its sacramental character have been equally misunderstood alike by those who profess to explain the one or to respect the other. The present work claims attention because it has been written by an intelligent woman, of scientific attainments, who has been married herself, and who professes to write of, and for, normal people only. This combination is very rare. Thus qualified, what, then, has Dr. Stopes to tell us? Put briefly, and therefore inadequately, she contends that the principal cause of unhappiness in marriage is ignorance of the art of love. The act of conjugation, which should be the joyful consummation of this relation, an act whose reactions colour, enrich, or poison the whole of the two lives whose union it completes, is too often a source of misery to one or to both. The reason given for this is neglect of the fact that while man's desire is practically constant, woman's has a marked tidal flow. The analysis of this flow is the book's original contribution. From a study of many cases Dr. Stopes believes that the normal woman is subject to a law of periodicity of recurrence of desire, whose rhythm normally reaches its crest just before, and about eight days after, menstruation. In other words, that the monthly rhythm of the latter is crossed or accompanied by a more or less conscious fortnightly rhythm of desire. If this is so, it is clear that conjugation can only be a joy to her, and, consequently, a mutual joy, when it coincides with this period. At other times it will be repulsive to her, if not worse. This is the crude statement of the theory which is treated with great subtlety and delicacy in the course of the book. Further, the writer relates to this the common fact that the period of excitation is much more speedy in the man than in the woman, and attributes to man's ignorant disregard of woman's physiology in this respect the relative rareness of mutual orgasm. She further suggests that the intervals between each fortnightly recurrence, while not too long for the average man's self-control, are long enough to maintain his desire unblunted. The effects of such intervals, restraint, and maintenance on the spiritual relation of marriage are suggestive to all, and convincing to those capable of apprehending them. On neglect of the facts implied by the above theory she explains the chief cause of unhappiness in married life: the root of much misery is there. There is an excellent chapter on "Sleep," wherein she points out that the sleep which naturally follows a mutally complete conjugation is not only the refreshing complement of the act itself, but is, as it were, the test and proof of its healthy accomplishment. Where this sleep is not mutual one of the pair has neglected the subtle laws to which she refers. We do not recall such an understanding of nuptial sleep anywhere save in Rossetti's beautiful, suppressed, sonnet with that title. In saying that, unless the art of love is studied, marriage cannot bear its full fruits, she sees, as the greatest thinkers have always seen, that marriage is a symbol of transcendental significance. There is more in the relation of Eros and Psyche than is dreamed of even in Harley Street. In later chapters Dr. Stopes discusses other influences in marriage, some highly controversial; but she wisely argues for those delicate reservations which alone can make marriage, in no rhetorical sense, a life-long voyage of discovery. Here she is not vague, or hazy. Every instance which she gives is concrete and direct with an engaging delicacy of frankness. When, however, she says that marriage has "never yet found a poet to sing its full glory," she has forgotten the great and neglected name of Coventry Patmore. In his "Angel in the House" she will find, perhaps to her surprise, many of the truths which she describes in terms of natural laws elaborated with astonishing subtlety and knowledge in the language of poetry. Her doctrine that after, as before, marriage, *every* "winning should be preceded by a wooing," is expounded by Patmore with the richest illustration. The present writer could give quotation after quotation did space permit. It is true that the dinner-bell rings too often in the poem, but in the "preludes" which form half of it, in the "wedding sermon" with which it closes, and, on the transcendental plane, in the "Unknown Eros," which was its after-song, many truths which Dr. Stopes recognises are implicit or expounded; explicit, indeed, for those who, like herself, have the root of the matter in them. In exquisite quality of understanding the book is a contribution to its subject, which in time will receive the recognition that it deserves.

**The Art of Keeping Well.** By Ronald Campbell Macfie, M.A., M.B. (London: Cassell and Co., Ltd. 1918. Pp. 248 + viii. Price 6s. 8d. net.)

There is not much opportunity for originality in the compiling of a book dealing with the subject of health from a popular point of view. The thing has been done over and over again, and, whatever may be the case with others, the jaded reviewer at least is heartily tired of the performance. Cheap and superficial explanations of physiological processes, arguments from half-valid analogies, gracious efforts to make things easy for the simple reader, and assurances that if we are good we shall be happy, are the stock-in-trade of this type of book. Dr Campbell Macfie has followed these well-established lines and the result does not differ materially from that produced by his predecessors. For those who like this kind of book there are all the seductions which flatter the amateur that he is in immediate contact with the innermost moods and mysteries of science. The suggestion is that the book is specially planned for "people under the strain of war conditions." Of course, we accept the motive thus declared, but we are bound to add that while the products of war and peace are in most respects widely different, the present volume so closely resembles those produced in more peaceful times that, but for the declaration on the cover, we should not have guessed that it had been born under the stress of an international conflict.

**The Battle with Tuberculosis and How to Win It.** A Book for the Patient and His Friends. By D. Macdougall King, M.B. Pp. 253, with 8 illustrations. (Philadelphia and London: J. B. Lippincott Company. Price 6s. net.)

A manual of instruction and encouragement for the consumptive (and a very American one, quoting Ella Wheeler Wilcox and other rhymesters indifferently with Marcus Aurelius). But it is a shame to laugh at it, for it is fluently written, and if the author can talk as well to patients, and if—important qualification—he can be as nasty to them when they break rules as he is here nice to them, he should, being a "cure" himself, be an ideal sanatorium superintendent. Very little to take exception to; but any pyrexia, however slight, means absolute rest in bed—that is fundamental. A good book for the social worker to read (better for him than for the patient, perhaps) or to put in the public library.

